# The effects of COVID-19 on rural communities in Mahikeng Local municipality

**DOI:** 10.4102/jamba.v16i1.1629

**Published:** 2024-07-23

**Authors:** Blessing Magocha, Mokgadi Molope, Martin Palamuleni

**Affiliations:** 1 Department of Population and Health Research Entity, Faculty of Humanities, North-West University, Mahikeng, South Africa; 2 Department of Development Studies, Faculty of Humanities, North-West University, Mahikeng, South Africa

**Keywords:** accessibility, affordability, availability, COVID-19, commodities

## Abstract

Global pandemics are known to disturb livelihoods. The coronavirus disease 2019 (COVID-19) is an example of such pandemic in the recent past. Its outbreak prompted a global response characterised by unprecedented measures to mitigate its spread. Several preventative measures were recommended by the World Health Organization (WHO) such as lockdowns to curtail the transmission of the virus and manage the crisis it caused. These measures hampered the movement and distribution of basic commodities inadvertently triggering a series of socio-economic consequences particularly in rural areas. This study delves into the intricate interplay between the COVID-19 lockdown and its impact on the accessibility, affordability and availability of basic commodities within the context of the Mahikeng Local Municipality, in a rural setting. Quantitative survey data were collected from 260 households, which were randomly selected. The data analysis was performed using chi-square, with a significance level of *p* < 0.05. The results showed that basic commodities were largely available (99%) in the market during lockdown. There was no significant association between background variables and availability of basic commodities. Accessibility of basic commodities was affected by many factors such as concerns of COVID-19 outbreak. The study demonstrated that prices of basic commodities increased during lockdown, thereby having a ripple effect on accessibility and affordability of basic commodities. However, the availability of basic commodities was less affected.

**Contribution:**

The case study approach, focusing on Mahikeng Local Municipality, is essential for capturing localised nuances and providing actionable insights to policymakers, researchers and community leaders seeking to mitigate the negative effects of lockdowns on rural populations.

## Introduction

The outbreak of the novel coronavirus disease (COVID-19) in late 2019 prompted a global response characterised by unprecedented measures to mitigate its spread (Béné et al. 2021; Kang et al. 2021). Among the most significant interventions were nationwide lockdowns and movement restrictions imposed by governments worldwide. While these measures aimed to curtail the transmission of the virus, they inadvertently triggered a series of socioeconomic consequences (Béné et al. 2021; Kang et al. 2021), particularly in rural areas where vulnerabilities were exacerbated (Anakpo, Hlungwane & Mishi 2023; Carlitz & Makhura 2021). This study delves into the intricate interplay between the COVID-19 lockdown and its impact on the accessibility, affordability and availability of essential commodities within the context of the Mahikeng Local Municipality, a representative rural setting, with the main objective of determining factors influencing the availability of basic commodities during the COVID-19 pandemic lockdown in Mahikeng Local Municipality. Furthermore, this study examined the predictors of accessibility and affordability of basic commodities during the COVID-19 pandemic lockdown.

Rural regions often possess unique socioeconomic dynamics that distinguish them from urban counterparts (Đurić, Simin & Glavaš-Trbić 2023; Kang et al. 2021). These socioeconomic dynamics are multifaceted, encompassing issues of limited infrastructure, reduced market access and lower income levels (Anakpo et al. 2023; Bordi et al. 2021; Đurić et al. 2023). This leads to geographic and economic isolation (Béné 2020) of the rural areas. The COVID-19 lockdown measures, while essential for public health, introduced a range of disruptions to these already fragile systems (Anakpo et al. 2023; Carlitz & Makhura 2021). This investigation focuses on Mahikeng Local Municipality as a microcosm of the broader rural landscape, aiming to uncover the nuanced repercussions of lockdown policies on the food security of its residents.

The existing literature emphasises that vulnerable populations, such as low-income households, elderly individuals and those with pre-existing health conditions, are disproportionately affected by the COVID-19 lockdown measures (Aaron et al. 2021; Kang et al. 2021; Louie, Shi & Allman-Farinelli 2022). These populations may face compounded challenges related to accessibility, affordability and availability of essential commodities (Aaron et al. 2021; Louie et al. 2022). Significantly, people living in rural areas and other marginalised settings were found to be experiencing marginal, low or very low food security than their counterparts (Louie et al. 2022). Food stocks were less available in urban areas than in rural areas in Bangladesh (18.8% vs. 37.8%), India (91.5% vs. 76.0%) and Myanmar (72. 0% vs. 59.0%) (Kang et al. 2021).

Scholarly work suggests that targeted policy interventions are crucial to address the adverse effects of lockdowns on rural areas (Kang et al. 2021). These interventions may include supporting local food production, improving transportation infrastructure, implementing social safety nets and fostering collaboration between local governments and communities (Kang et al. 2021). Kang et al. also recommended strategies to target different aspects of livelihoods in urban and rural areas so as to maximise the resilience of local food systems. Shahzad et al. (2022) recommends that the problem of food inaccessibility during COVID-19 could be solved by designing strategies that are reflective of the changed people behaviour and innovative market services such as advancement of technology to promote e-commerce.

This study focuses on only two (availability and access) of the four pillars of food security: availability, access, utilisation and stability (Béné et al. 2021; Kang et al. 2021). Gathering comprehensive data on all four food security pillars was challenging during a rapidly evolving crisis. Hence, the focused approach was considered desirable for a more comprehensive analysis of key issues without diluting the discussion with additional factors. Accessibility has been divided into two, that is economic (affordability) and physical accessibility (Béné et al. 2021). Accessibility, a fundamental aspect of this study, addresses the ease with which residents can obtain necessary commodities and services. Lockdown-induced restrictions on movement, coupled with reduced public transportation services, could potentially hinder the ability of rural residents to reach markets and shops. The study seeks to explore how changes in mobility patterns during the lockdown influenced residents’ access to essential commodities and whether certain demographic groups were disproportionately affected.

Affordability, another critical dimension, pertains to the economic ability (Béné et al. 2021) of rural inhabitants to purchase essential goods and services. The economic downturn resulting from the pandemic, with its associated job losses and income reductions, has the potential to impact household capacity to purchase food items for consumption (Kang et al. 2021). This study investigates whether the pandemic and its subsequent lockdown exacerbated existing disparities in affordability within the Mahikeng Local Municipality and how these changes have influenced consumption patterns.

Availability of commodities, the focus of this study, refers to the consistent presence of essential goods in local markets (Onyeaka et al. 2022). Supply chain disruptions, both global and local, are induced by lockdown restrictions and could lead to shortages or uneven distribution of commodities (Guan et al. 2020; Gupta & Jiwani 2020). The study aims to examine shifts in the availability of goods in Mahikeng’s markets. By focusing on the specific case of Mahikeng Local Municipality, this research endeavours to generate insights that can contribute to a broader understanding of the intricate relationships between pandemic-induced lockdowns and rural economies. The findings from this study are expected to inform policymakers, researchers and community leaders about the measures required to strengthen rural resilience in the face of future crises. The research underscores the importance of proactive and targeted interventions to safeguard the well-being of vulnerable rural populations during times of global uncertainty. The significance of this study is multifaceted and extends to many stakeholders and the broader society. Firstly, it fills in the data gap that exists in Mahikeng and many rural areas in South Africa and beyond regarding the food security during the COVID-19 lockdowns (Arndt et al. 2020; Ngarava 2022; Wegerif 2020). It also provides concrete insights into how lockdown measures impacted rural households in Mahikeng Local Municipality’s ability to access basic commodities. Understanding the challenges faced by rural areas such as Mahikeng can help inform the development of targeted policies and interventions that address specific issues arising from such situations in the future. Hence, the study’s findings may provide empirical evidence that informs decision-making processes related to food security and sustainable development.

Apart from that, the findings can contribute to enhancing the preparedness and resilience of rural areas against future crisis. Local authorities and organisations can benefit from the study’s insights to allocate resources more effectively during emergencies. This can involve directing support towards improving infrastructure, strengthening supply chains and implementing strategies that enhance accessibility and affordability of basic commodities for rural households. The study’s findings can contribute to understanding the long-term economic consequences of lockdown measures. The study is informed by the theory of structural functionalism presented in the following section.

## Theory of structural functionalism

The theory of structural functionalism, proposed by sociologist Emile Durkheim and later developed by Talcott Parsons, emphasises the interdependence and equilibrium within societies (Harper 2011; Kingsbury & Scanzoni 1993). This theory suggests that societal components (structures) work together to maintain stability and functionality (Harper 2011). When applied to this study, this theory provides insights into how rural communities, such as Mahikeng Local Municipality, respond to and adapt during the COVID-19 lockdown to maintain their overall functionality. In the context of the COVID-19 lockdown’s effects on rural areas, structural functionalism can help explain how different aspects such as local markets, government social safety nets and community organisations interact to cope with the challenges posed by the pandemic and its subsequent restrictions. The theory highlights that societies are composed of various interconnected institutions, each serving specific functions to ensure the smooth operation of the whole. Among them, local markets, transportation networks, community organisations and government agencies have responded to the lockdown measures. However, the theory of structural functionalism tends to be a community-based theory and focuses on how communities when faced with a pandemic survive. This study covers the gap on how community members and households adapt to survive during a pandemic with reference to the COVID-19. We examined how these structures collaborated to ensure the accessibility, affordability and availability of basic commodities for the rural people in Mahikeng Local Municipality.

## Research methods and design

### Study design

The study adopted a cross-sectional survey study design. This involves collecting data from respondents only once at a specific time. Cross-sectional surveys are not expensive as the researcher does not follow up individuals over time; however, it is difficult to establish causal relationship from such studies (Connelly 2016). The questionnaire was administered from November 2022 to December 2022.

### Study setting and population

Two rural villages (Tsetse and Miga) in Mahikeng Local Municipality were purposively sampled for the study because of their proximity to the university. The Mahikeng municipality is a bigger local municipality compared to the other four local municipalities located in Ngaka Modiri-Molema District (DSSA 2022) in the North-West Province of the Republic of South Africa (RSA). It is divided into 35 wards consisting of 102 villages and suburbs (COGTA 2020), with an estimated population of 305 291 people. Approximately 75% of the area is rural and under the tribal authority (DSSA 2022). Tsetse village has a total population of 4003 and 1062 households, while Miga village has 2046 people and 736 households, respectively (DSSA 2022).

### Study sampling strategy

A multistage sampling design was employed in this study. The first stage of the sampling employed the purposive sampling technique to select two villages from Mahikeng Local Municipality. They were chosen on account of resources and COVID-19 regulations, which prevented extended travelling as well as people’s willingness to participate in the study. Out of a total of 1798 households, 260 were randomly selected. After that, we applied a systematic sampling strategy to select the actual households to complete the questionnaire. Each household was given a number from 1 to 260. We selected a random number for the first household and thereafter we visited every fifth household. The list of these households was secured from DSSA website as of 02 February 2022 (DSSA 2022). The sample size was calculated using the following equation adopted from Fisher formula (Fisher 1998):

$$n=\frac{\frac{z^{2}P(1-P)}{d^{2}}}{1+\frac{1}{N}\left(\frac{z^{2}P(1-P)}{d^{2}}-1\right)}$$ [Eqn 1]

Where:

Confidence level= 95%

z-score = 1.96

Precision +/- = 5%

Population size = 1798

Assumed P = 20%

Sample size (*n*)= 217 households.

Note: It was assumed that the livelihoods of 20% of the households in the villages were affected by the COVID-19 pandemic based on the earlier estimates. Therefore, this study adopted the 20% as the P (proportion) affected by COVID-19 from previous study (Jain et al. 2020). This assumption is based on the findings of National Income Dynamics Study (NIDS) – Coronavirus Rapid Mobile Survey (CRAM), the first of its kind ever conducted in the country. The survey interviewed 7074 individuals aged between 18 and 64 (Jain et al. 2020). Therefore, the sample size of 217 households was adjusted by 0.20 non-response rate to cater for non-availability of sampled unit. Finally, the sample size was 260 households selected for the study. Probability proportional to size (PPS) sampling was used. In village 1, 153 households were selected and 107 in village 2. As illustrated in Table 1.

**TABLE 1:** Probability proportional to size sampling.

Village	Calculated sample	Target population	Proportion	Sample per village
Village 1	-	1062	0.59	153
Village 2	-	736	0.41	107

**Total**	**260**	**1798**	**1**	**260**

*Source:* DSSA, 2022, *Census: Improving lives through data ecosystems*, Republic of South Africa, Pretoria

The study selected only head of households aged 18 and over who consented to complete the questionnaire.

### Data collection procedure

A questionnaire was used to collect data from the prospective respondents on the effect of COVID-19 on accessibility, affordability and availability of basic commodities during the pandemic. The questionnaire consisted of a consent form and a household roster, which was used to collect household information. The questionnaire also collected background information (household demographics).

## Measures of outcome variable

Three dependent variables: availability of basic commodities, accessibility of basic commodities and affordability of basic commodities were examined in the study. Availability of basic commodities were measured using the following question: ‘were basic commodities available in the market during the COVID-19 lockdown?’ And the following responses were provided (Yes or No). Accessibility of basic commodities was measured to see whether basic commodities were accessible in the market during the COVID-19 lockdown. The respondents were asked to select options that best represent their opinion regarding their households, ‘my household failed to access food from the market between March and April 2020 because’, the following variables were provided: market or grocery stores were closed; transport limitations; movement restrictions; security concerns; concerned about leaving the home because of COVID-19 outbreak; members of the household were unwell; no food in the market or grocery stores; little or no money to buy food. The following responses were provided for each variable: strongly disagree, disagree, somewhat disagree, agree and strongly agree. Lastly, affordability was measured to see whether the food prices were affordable. The question ‘did food prices increase during the COVID-19 lockdown?’ was asked and the responses were (Yes or No).

## Measures of explanatory variables

### Background variables

The demographic factors were as follows: age (< 20, 20–29, 30–39, 40–49, 50–59, 60+); sex (male, female); level of education (primary, secondary, higher, none); place of residence (Tsetse, Miga); marital status (married, formerly married, never married); employment status (yes, no); source of income (salary with regular income, informal employment, remittances, government assistance) and household size (1–2, 3–4, 5+).

### Data analysis

Frequency distributions were used to describe and summarise the characteristics of the respondents. In addition, the bivariate relationship between the background characteristics and the dependent variables were examined using the chi-square test of independence set at *p* < 0.05 to show whether there was an association between background characteristics of the respondents and the dependent variables (accessibility and availability) of basic commodities during COVID-19 lockdowns. Two bivariate analysis (accessibility and availability) were computed to determine the statistically significance level set at *p* < 0.05.

### Ethical considerations

Ethical approval of the study was obtained from the Basic and Social Sciences Research Ethics Committee (BaSSREC) on the 24/08/2022. Permission was granted to conduct research by the North-West University Senate Committee for Research Ethics (NWU-SERC) under ethics number NWU-01008-22-A7. In addition, permission was also sought from the chiefs and gatekeepers to access the two villages. The identified respondents consented to respond to the questionnaire in private places. The consent form that they completed outlined the purpose of the study as well as the fact that participation is voluntary and can be withdrawn whenever they so wish without any consequences. Confidentiality was observed and no names of respondents were recorded; only numbers were used as questionnaire signifiers.

## Results

### Background characteristics

Table 2 represents the frequency distribution of background characteristics of the respondents. Respondents aged 60+, dominated the sample (28%), whereas the respondents < 20 years had only (2%). The majority of the respondents (70%) were females. A significant proportion of the sample reported being educated. Thus, majority of the respondents reported having secondary education (62%), whereas only (20%) of the respondents reported having no education. With regard to place of residence, (59%) of the sample reported living in tsetse, whereas 41% of the sample belonged to Miga residential places. More than half of the sample, 56% reported having never been married while a few respondents reported having been formerly married (19%). A significant proportion of the sample, (85%) reported being employed. Majority of the sample (55%) reported government assistance or social safety nets as their source of income, whereas only 14% reported being informally employed and on salary as their regular source of income. Lastly, the majority of the respondents (61%) reported their household size being 1–2 people. Only a handful proportion of the respondents (6%) reported their household size being 5+ people.

**TABLE 2:** Sociodemographic characteristics of the respondents (*N* = 260).

Variable	Frequency	Percentage
**Age (years)**		
< 20	4	1.5
20–29	37	14.0
30–39	62	23.5
40–49	44	16.7
50–59	43	16.3
60+	74	28.0
**Sex**		
Male	79	29.9
Female	185	70.1
**Level of education**		
Primary	59	22.3
Secondary	164	62.1
Higher	21	8.0
None	20	7.6
**Place of residence**		
Tsetse	156	59.1
Miga	108	40.9
**Marital status**		
Married	66	25.0
Formally married	50	18.9
Never married	148	56.1
**Employment status**		
Yes	39	14.8
No	225	85.2
**Source of income**		
Salary with regular income	38	14.4
Informal employment	36	13.6
Remittances	45	17.0
Government assistance	145	54.9
**Household size**		
1–2	162	61.4
3–4	86	32.6
5+	16	6.1

### Availability

Food availability is important to achieve the sustainable development goal number 2, which advocates for zero hunger for all. Supply chain disruptions, both global and local, induced by lockdown restrictions could lead to shortages or uneven distribution of commodities. In this study, the largest proportion of the sample (99%) reported basic commodities being available during the COVID-19 pandemic period.

The study determined the influence of background characteristics on availability of basic commodities during COVID-19 pandemic period (see Table 3). There was no significant association between age, sex, education, place of residence, marital status, employment status, source of income and household size by availability of basic commodities during the COVID-19 pandemic period.

**TABLE 3:** Percentage distribution of availability of basic commodities during the coronavirus disease 2019 lockdown period by background variables (*N* = 260).

Variable	Percentage	*P*
**Age (years)**	-	0.642
< 20	100.0	-
20–29	97.3	-
30–39	100.0	-
40–49	100.0	-
50–59	100.0	-
60+	98.6	-
**Sex**	-	0.354
Male	100.0	-
Female	98.9	-
**Level of education**	-	0.142
Primary	100.0	-
Secondary	99.4	-
Higher	100.0	-
None	95.0	-
**Place of residence**	-	0.793
Tsetse	99.4	-
Miga	99.1	-
**Marital status**	-	0.454
Married/ living together	100.0	-
Formerly married	100.0	-
Never married	98.6	-
**Employment status**	-	0.555
Yes	100.0	-
No	99.1	-
**Source of income**	-	-
Salary with regular income	100.0	-
Informal employment	100.0	0.647
Remittances	100.0	-
Government assistance	98.6	-
**Household size**	-	0.530
1–2	98.8	-
3–4	100.0	-
5+	100.0	-

### Accessibility

The study further assessed the accessibility of basic commodities from the market during the COVID-19 pandemic lockdown period (Table 4). Accessibility, a fundamental aspect of this study, addresses the ease with which residents can obtain necessary commodities and services. Among those who reported failure because of market or grocery stores being closed, majority of the respondents, 36% strongly disagreed. Furthermore, a sizeable proportion of the respondents, 31% strongly agreed to transport limitations enabling their failure to access market spaces during the COVID-19 pandemic period. More so, a handful proportion of respondents (29%), reported strongly disagreeing to movement restrictions causing their failure to access market spaces to secure basic commodities.

**TABLE 4:** Frequency distribution of the respondent’s accessibility to market spaces during the coronavirus disease 2019 pandemic period (*N* = 260).

Variable	Strongly disagree	Disagree	Somewhat disagree	Agree	Strongly agree
**My household failed to access basic commodities from the market during the COVID-19 lockdown period**					
Market/grocery stores were closed	36.0	14.8	18.6	20.5	10.2
Transport limitations	26.9	15.5	5.7	20.5	31.4
Movement restrictions	28.8	14.0	5.7	23.1	28.4
Security concerns	31.8	11.4	2.3	18.2	36.4
Concerned about leaving the home because of COVID-19 outbreak	33.7	5.7	2.7	12.9	45.1
Members of the household were unwell	72.3	14.0	7.6	0.0	6.1
No food in the market or grocery stores	53.4	15.5	10.6	14.4	6.1
Little or no money to buy food	26.5	6.4	7.2	23.9	36.0

Further analysis revealed that 36% respondents strongly agreed that security concerns resulted in their failure to access markets or grocery stores. A large proportion of respondents (45%) reported they failed to access markets to buy basic commodities because of concerns of leaving home as a result of of the COVID-19 outbreak. Majority of the respondents (72%) reported strongly disagreeing to failure to access market spaces as a result of members of the household who were unwell. A larger proportion of the respondents (53%) reported failure to access market spaces because of no food in the market or grocery stores. Lastly, a sizeable proportion of respondents (36%) reported failure to access market spaces because of little or no money to buy food.

### Affordability

**FIGURE 1:**
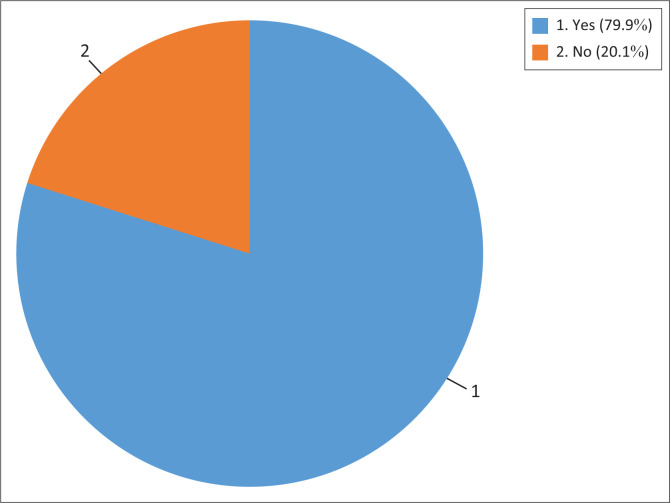
Frequency distribution of price increase for commodities during the coronavirus disease 2019 pandemic (*N* = 260).

The study further examined the affordability of the commodities during COVID-19. Hence, this study examined the increases of food price during COVID-19 lockdown (see Figure 1). The result found that majority of the respondents (80%) reported an increase in the price of basic commodities during the COVID-19 pandemic period, whereas 20% reported no increase.

Food price changes at the local level were affected by the dynamics of the COVID-19 pandemic, with some countries witnessing steep food price increases (Shahzad et al. 2022). The study further conducted a chi-square test of independence to determine the relationship between increase in prices of commodities by background variables during the COVID-19 pandemic period (see Table 5). Hence, there was no significant association between age, sex, education, place of residence, marital status, employment status, source of income and household size by increase in price of basic commodities during the COVID-19 pandemic period.

**TABLE 5:** Percentage distribution of increase in price of basic commodities during the coronavirus disease 2019 lockdown period by background variables (*N* = 260).

Variable	Percentage	*P*
**Age (years)**	-	0.538
< 20	75.0	-
20–29	83.8	-
30–39	72.6	-
40–49	84.1	-
50–59	86.0	-
60+	78.4	-
**Sex**	-	0.962
Male	79.7	-
Female	80.0	-
**Level of education**	-	0.869
Primary	83.1	-
Secondary	79.3	-
Higher	81.0	-
None	75.0	-
**Place of residence**	-	0.831
Tsetse	79.5	-
Miga	80.6	-
**Marital status**	-	0.995
Married/living together	80.3	-
Formerly married	80.0	-
Never married	79.7	-
**Employment status**	-	0.220
Yes	87.2	-
No	78.7	-
**Source of income**	-	0.242
Salary with regular income	81.6	-
Informal employment	91.7	-
Remittances	80.0	-
Government assistance	76.6	-
**Household size**	-	0.512
1–2	80.9	-
3–4	80.2	-
5+	68.8	-

## Discussion

This study delves into the intricate interplay between the COVID-19 lockdown and its impact on the accessibility, affordability and availability of basic commodities within the context of the Mahikeng Local Municipality in a rural setting. It examined the accessibility, affordability and availability of basic commodities in the market during COVID-19.

The study found that a larger proportion of the respondents (53%) could not access market space because there was no food on the market or grocery stores. This could have been necessitated by panic buys and food hoarding that took place soon after the lockdowns were pronounced. A contradictory finding was made in a rapid assessment survey (Kang et al. 2021) conducted among Non-Governmental Organisations that supported communities in six Asian countries which found out that food and essential items were largely available in the markets without significant difference between rural and urban areas except in Vietnam. However, Béné et al. (2021) concluded that accessibility of food was the most affected dimension of people’s food security, and this was as a result of both financial component (affordability) following job and income losses and physical access to food outlets especially during the initial periods of complete lockdown. This is possible considering that in places such as Punjab and Sindh in Pakistan 98.78% sample of a population in study reported that they were quarantined in their homes (Shahzad et al. 2022). Complete lockdowns resulted in loss of customers for some of the consumable goods with some producers resorting to pouring away unsold milk in Bangladesh (Hossain 2020). The same was witnessed in America where producers found themselves with surplus of food without the ability to distribute it to market and some resorted to destroying it or reduce capacity at processing plants (Cable et al. 2021). Cable et al. (2021) further highlighted that globally the immediate impact of the COVID-19 on the food supply chain was largely logistical and food stocks were more than sufficient because of the previous year’s harvest.

The study also found out that transport limitations necessitated failure to access market spaces during the COVID-19 pandemic period. This corroborates the results of studies performed elsewhere (Cable et al. 2021; Jafri et al. 2021). However, in another study, they found that physical access to markets or neighbourhood kiosks for basic food items was not hindered in Bangladesh, Indonesia and Myanmar (Kang et al. 2021). This in some sense contradicts the results of Béné et al. (2021) who found out that formal outlets thrived, that is the home delivery, grocery stores and supermarkets at the expense of informal outlets that were closed as they were not considered essential services.

The findings of this study highlight that the prices of basic commodities increased in Mahikeng Local Municipality. The result found that majority of the respondents (80%) reported an increase in the price of basic commodities during the COVID-19 pandemic period. This led to economic inaccessibility of basic commodities in the rural areas. Results of studies carried out elsewhere suggest that the economic downturn triggered by the pandemic and lockdowns has led to job losses, income reductions (Kang et al. 2021), which caused decline in purchasing power of households in LMICs (Béné et al. 2021; Carlitz & Makhura 2021) and increased financial stress, particularly in rural areas. This has a direct impact on the affordability of commodities for rural households, potentially leading to food insecurity and decreased well-being. Kang et al. (2021) found out that poor affordability of essential expenses was particularly pronounced for loan repayments and rent payments, compared to food. This same result was found in Vietnam where rent and loan payment were least affordable among the groups that were interviewed (Aaron et al. 2021). A pathway analysis by Béné et al. (2021) confirmed this as the central impact of the degradation in food affordability on people’s food security. However, they suggested that the degradation was not because of rise in food stuff prices but from a decline in purchasing power at the consumer level (Béné et al. 2021). The same was noticed in South Africa where households resorted to reduced spending to compensate for loss of income and some used their savings (Carlitz & Makhura 2021). In contrast, Shahzad et al. (2022) found out that affordability was affected by price increase as highlighted by about 91.56% of the people who participated in the survey conducted in Punjab and Sindh provinces in Pakistan. Interestingly, a study by Hossain (2020) found out that in Bangladesh some food stuffs were sold below the production cost because of lockdowns, for example, poultry meat, which was sold at BDT70 per kg, while the cost of production was estimated to be BDT100 per kg. An increase in commodity prices was also noticed in study performed in US, which compared 2019 and 2020 prices, for example, they found out that egg prices increased 38% for April 2020 compared with 2019 and ground beef increased 11% and whole milk increased 10% in the same period (Cable et al. 2021).

The findings show that basic commodities were available in the market in Mahikeng Local Municipality. The majority (99%) reported that basic commodities were available during COVID-19 pandemic. However, studies carried out elsewhere have shown that supply chains for essential goods in rural areas have been disrupted because of transportation limitations and closures of manufacturing facilities (Cable et al. 2021). This has resulted in shortages and uneven distribution of commodities, affecting the availability of goods in local markets. In US, uneven distribution of commodities caused shortages of some commodities at grocery stores such as meat (Cable et al. 2021). In a study by Kang et al. (2021), it was found that essential items such as basic foods and fresh foods were available in the six countries that they studied, which indicates that the market system was not severely affected by lockdown (Kang et al. 2021). Béné et al. (2021) concur that function of food systems to supply food did not collapse and continued to work and delivered food throughout 2020. Hence, this can best explain the results of study in Vietnam, which found out that starch products were widely available to both the less impacted (94.3%) and the fully impacted or severely impacted group (93.0%) (Aaron et al. 2021). However, a study performed by Béné et al. (2021) found that 21% of households in Nepal identified shortages of food in markets and food outlets as the main reason for insufficient food. In contrast, Jafri et al. (2021) who conducted a cross-sectoral multi-country online survey found out that lockdowns triggered shortages of staple foods because of bulk buying and stockpiling.

## Conclusion and recommendations

The COVID-19 pandemic brought about extraordinary disruptions to economies, societies and daily life worldwide. Rural communities, often characterised by limited access to resources and services, faced diverse challenges during lockdowns. The lockdown because of COVID-19 had adverse consequences on accessibility and affordability of basic commodities in Mahikeng Local Municipality. The study demonstrated that prices of basic commodities increased during lockdown by having ripple effect on accessibility and affordability of basic commodities. However, availability of basic commodities was less affected. The findings underscore the multifaceted impacts of COVID-19 lockdown on the accessibility, affordability and availability of commodities in rural areas. The case study approach, such as the one focusing on Mahikeng Local Municipality, is essential for capturing localised nuances and providing actionable insights to policymakers, researchers and community leaders seeking to mitigate the negative effects of pandemic-related restrictions on rural populations. The findings provide a reconstruction of how households in Mahikeng Local Municipality’s food security were affected during the initial phases of the COVID-19 pandemic and lockdowns. The study recommends formulation of strategies to target different aspects of livelihoods in rural areas to maximise the resilience of local food systems, such as market gardening. There is a need for further research that uses other models of measuring food security such as the Food Insecurity Experience Scale (FIES) or Household Food Security Access Prevalence (HFIAP) in conjunction with the traditional measures of food consumption, dietary quality and nutritional status.
